# Persistent Atelectasis Prevalence and Incidence in Pediatric Critical Care

**DOI:** 10.7759/cureus.83888

**Published:** 2025-05-11

**Authors:** Amani Nabri, Abdulaziz Alshehri, Sarah A Alfurayj, Zahra M Alshakhs, Afnan N Alshayeb, Rawan Alhammadi, Ghadh A Alenazi, Razan Bokhari, Rand B Ashoor

**Affiliations:** 1 College of Medicine, Dar Al Uloom University, Riyadh, SAU; 2 Respiratory Therapy, King Fahad Medical City, Riyadh, SAU

**Keywords:** atelectasis, critical care, incidence, pediatrics, prevalence

## Abstract

Background and objective

In critical care, persistent atelectasis worries pediatricians. The research on hospitalized pediatric patients with persistent atelectasis reports limited data about prevalence and incidence. The study aimed to determine the prevalence and incidence of persistent atelectasis in pediatrics.

Methodology and materials

This retrospective observational study was carried out in the pediatric intensive care unit (PICU) of King Fahad Medical City (KFMC) between February 2020 and October 2023. Pediatric patients with pulmonary atelectasis for more than 48 hours. Patients who were admitted to the PICU were taken into the study and evaluated after taking informed consent. Patients who were prescribed dornase alfa for atelectasis treatment. The prevalence and incidence of the disease were calculated in both treatment groups; independent t-tests and chi-square tests were used to compare continuous and categorical variables, respectively, between the dornase and non-dornase groups. Correlation analysis was performed using Pearson’s correlation coefficient to examine relationships between clinical parameters. Regression analysis was conducted to identify significant predictors of intensive care unit (ICU) length of stay and persistent atelectasis, with model significance evaluated using the F-statistic and R² values. A p-value < 0.05 was considered statistically significant for all analyses.

Results

The prevalence of persistent atelectasis reached 48.18% among patients who received dornase, while the non-dornase group experienced 51.82% atelectasis occurrence. The incidence analysis demonstrated a major distinction between groups where dornase-treated patients experienced 26.4 new at-risk patient cases but non-dornase-treated patients developed 71.5 new cases.

Conclusion

The dornase treatment group demonstrates the potential to decrease new persistent atelectasis incidence in pediatric critical care units. The non-dornase group showed slightly higher persistent atelectasis prevalence and incidence rates yet dornase treatment seemed to prevent atelectasis progression in pediatric patients at risk. The research demonstrates dornase's ability to prevent atelectasis occurrence yet its effect on patient overall survival remained concerning because of the retrospective study design. Future researchers are encouraged to do studies that help to establish a causal relationship between dornase alfa and persistent atelectasis occurrence.

## Introduction

In pediatric critical care, persistent atelectasis represents a major concern because it causes lung tissue to remain partially collapsed [1]. Inadequate gas exchange from atelectasis causes respiratory distress along with longer hospitalization periods [2]. The condition of atelectasis affects critically ill children most frequently when they receive mechanical ventilation or have existing pulmonary diseases [3]. The prevalence and incidence rates of persistent atelectasis in pediatric critical care facilities remain unclear, along with its effects on future health outcomes in affected patients. Accelerated diagnosis processes and appropriate treatments for this condition lead to better clinical outcomes for children under critical care with simultaneous prevention of complications [4].

Volume loss from the collapse of a segment, lobe, or entire lung parenchyma is known as atelectasis [5]. Increased surface tension from airway blockage, parenchymal compression, surfactant insufficiency, or malfunction can lead to atelectasis [6]. Children are particularly susceptible to atelectasis due to their tiny airways, which are prone to collapse, and less frequent collateral ventilation [7]. The causes of atelectasis are neurological illnesses, cystic fibrosis, primary ciliary dyskinesia, and chronic lung diseases, including asthma and airway malacia. Early identification and treatment remain essential for children with neurological disorders because atelectasis presents a major cause of morbidity and mortality [8].

Research about persistent atelectasis in children remains scarce, while studies focusing on hospitalized children remain limited. The lack of research on persistent atelectasis prevalence and incidence in pediatric critical care needs additional studies to understand its effects on patient outcomes [9,10]. Healthcare providers struggle with effective prevention and management strategies because there is inadequate clinical data regarding persistent atelectasis characteristics, contributing factors, and long-term effects [8,10]. The study of persistent atelectasis requires investigation into its primary risk factors and evaluation of its medical consequences on patient health. Healthcare providers should develop improved clinical practices for patient care by studying persistent atelectasis prevalence data along with its impact on intensive care unit (ICU) stay duration and recovery time. The study evaluated atelectasis patterns in addition to patient statistics and related variables from children admitted to critical care units.

Objective

The primary objective of this study was to determine the prevalence and incidence of persistent atelectasis in pediatric patients admitted to the pediatric intensive care unit (PICU). The secondary objective was to identify demographic and clinical factors associated with persistent atelectasis and to assess the impact of the disease on patients’ outcomes, such as length of hospital stay and recovery times.

## Materials and methods

Study design and ethical consideration

A retrospective study was conducted including pediatric patients admitted to the PICU of King Fahad Medical City (KFMC). The study was carried out after obtaining approval from the Institutional Review Board, King Abdulaziz City for Science and Technology (KACST), Kingdom of Saudi Arabia (KSA) (Approval No. H-01-R-012). The anonymity and confidentiality of the patient’s data were assured. The data was handled only for analysis and research purposes for the benefit of patients. The data of the patients were de-identified before being used in research.

Study setting and sample size

The study was carried out on pediatric patients in the PICU between February 2020 and October 2023 using data from the KFMC medical database.

Study participant recruitment

The eligibility criteria of the patients enrolled in the current study were to include non-cystic fibrosis pediatric patients with or without respiratory support and persistent pulmonary atelectasis from February 16, 2020, to October 10, 2023. The patient was recruited using the electronic database of KFMC through the Electronic Patient Information Chart (EPIC) research module system.

Inclusion criteria

The following were the inclusion criteria: pediatric patients with pulmonary atelectasis for more than 48 hours, patients who were admitted to the PICU, and patients who were prescribed dornase alfa for atelectasis treatment.

Exclusion criteria

The following were the exclusion criteria: cystic fibrosis (CF) patients, patients who were not admitted to the PICU, patients with non-persistent pulmonary atelectasis (less than 48 hours), and patients with missing or incomplete data.

Data collection procedures and tools

The data was collected from the electronic database of KFMC through the EPIC research module system. The authors monitored patients who had lung collapse during their stay in the PICU for more than 48 hours and underwent treatment with dornase alfa via nebulization. However, all patients were treated with Ventolin 2.5 mg as standard therapy to bronchodilate and facilitate secretion removal. Also, other treatment modalities were collected, such as manual positive pressure ventilation and chest physiotherapy. Moreover, additional therapies targeting atelectasis, like Ventolin, Atrovent, 3% hypertonic saline, and acetylcysteine. Active diagnosis, chronic diagnosis, and disease background were documented as per the PICU daily rounds note. The data was divided into two groups: pediatric patients who were treated with dornase alfa and those who were treated with other treatments other than dornase alfa (non-dornase alfa group). All the data mentioned ahead were separately collected and added for both groups. Moreover, airway status, endotracheal size and level, and respiratory support (mechanical ventilator, high-flow nasal cannula, or noninvasive ventilation) were monitored along with the parameters of the ventilator, e.g., a fraction of inspired oxygen (FiO_2_), dynamic compliance, and positive end-expiratory pressure (PEEP), and changes were recorded within the time window of the atelectasis. Patient ICU length of stay in days, 28-day outcome, and other physiological parameters like central venous pressure and SpO_2_ (oxygen saturation)/FiO_2 _ratio were documented to determine the cost-effectiveness.

The electronic chest radiographs were taken, and an atelectasis assessment was conducted via the Modified Radiological Assisted Score (MRAS). The modified radiological atelectasis score: each lobe (including the lingula) is scored 0-3 (0=normal, 1=plate or minor infiltrate, 2=moderate atelectasis, 3=total atelectasis). The scores of the six lobes are then summed to give an 18-point score (0-18). The dynamic compliance was determined, such as the scores of 0.00-1.00 (severe), 1.01-2.50 (moderate), 2.51-5.00 (mild), 5.01-10.00 (normal), and 10.01 or above (increased compliance).

The prevalence was calculated using the following formula: Prevalence = (total persistent atelectasis cases in the dornase group/total dornase patients) x 100. However, the incidence was calculated using the formula (new cases of atelectasis in the dornase group/at-risk patients in the dornase group). 

The outcome measures of the study were improvement in atelectasis determined by MRAS before and after treatment (for calculating mean change). Moreover, the lung collapse resolution was computed using pre- and post-MRAS scores. If the post-MRAS score reduction was more than 50%, it was labeled as a complete resolution; for MRAS score changes around 50%, it was taken as partial resolution, and other patients with MRAS score changes of 1 or 2 or less than 50% improvement in MRAS score were labeled as no resolution. The collapse resolution outcome is cross-tabulated with PEEP change to determine that the increase in PEEP score played a role in the resolution of lung collapse. The data was handled carefully to ensure transparency and validity. 

Statistical analysis

IBM SPSS Statistics for Windows, Version 28 (IBM Corp., Armonk, NY) was used to analyze data. The statistical analysis for this study involved both descriptive and inferential methods to compare clinical variables and assess their associations with patient outcomes. Continuous variables were summarized using means and standard deviations, while categorical variables were presented as frequencies. The prevalence and incidence of the disease were calculated in both treatment groups; independent t-tests and Chi-square tests were used to compare continuous and categorical variables, respectively, between the dornase and non-dornase groups. Correlation analysis was performed using Pearson’s correlation coefficient to examine relationships between clinical parameters such as age, gender, status, airway status, endotracheal tube (ETT) level, number of affected lobes, collapse duration (days), central venous pressure (CVP), SpO_2_/FiO_2_, FiO_2_ (%), PEEP trends, PEEP changes, medications, secretion amount before treatment, secretion color before treatment, secretion amount after treatment, secretion color after treatment, 28-day outcome, endotracheal tube size, and cuff.

Regression analysis was conducted to identify significant predictors of ICU length of stay and persistent atelectasis, with model significance evaluated using the F-statistic and R² values. A p-value < 0.05 was considered statistically significant for all analyses.

## Results

Prevalence and incidence

The prevalence of persistent atelectasis in pediatric intensive care unit (PICU) patients was found to be 48.18% in the dornase group and 51.82% in the non-dornase group. While the prevalence suggests that dornase may have a role in atelectasis management, other contributing factors are likely influencing its persistence. The incidence analysis revealed a significant contrast between the two groups, with an incidence rate of 26.4 per at-risk patient in the dornase group compared to 71.5 per at-risk patient in the non-dornase group (Table 1).

**Table 1:** Prevalence and incidence of persistent atelectasis.

Prevalence for dornase and non-dornase groups
Prevalence = total persistent atelectasis cases in dornase group/total dornase patients × 100
Prevalence = 132/274 × 100 = 48.18%
Prevalence = total persistent atelectasis cases in non-dornase group/​total non-dornase patients × 100
Prevalence = 142/274 × 100 = 51.82%
Incidence for dornase and non-dornase groups
Incidence = new cases of atelectasis in dornase group/at-risk patients in dornase group
Incidence = 132/5 = 26.4
Incidence = new cases of atelectasis in non-dornase group/at-risk patients in non-dornase group
Incidence = 143/2 = 71.5

Table 2 presents a comparison of clinical variables between pediatric patients receiving dornase and those who did not in the PICU. The analysis revealed no significant difference in age (p = 0.620) or collapse duration (p = 0.064) between the two groups. However, central venous pressure (CVP) was significantly lower in the dornase group (2.93 ± 5.63) compared to the non-dornase group (4.87 ± 6.04, p = 0.020). Oxygenation parameters showed notable differences, with SpO_2_/FiO_2_ being significantly lower in the dornase group (p = 0.026), while FiO_2 _and PEEP-related measures did not differ significantly (p > 0.05). Importantly, ICU length of stay was significantly longer in the dornase group (25.63 ± 27.58 days) than in the non-dornase group (15.09 ± 18.37 days, p = 0.001). Additionally, MRAS scores before and after treatment were significantly higher in the dornase group (p = 0.001), though the change in MRAS also showed a significant improvement (6.08 ± 2.69 vs. 5.14 ± 2.45, p = 0.003). 

**Table 2:** Comparison of clinical variables between dornase and non-dornase groups. CVP: central venous pressure; SpO_2_: peripheral capillary oxygen saturation; FiO_2_: fraction of inspired oxygen; PEEP: positive end-expiratory pressure; MRAS: Modified Radiological Assisted Score; DNase: deoxyribonuclease; '-' or ---- indicates not applicable or not estimated.

Variable	Groups	N	Mean ± SD	t-statistics	p-value
Age (days)	Dornase	126	34.16 ± 41.31	-0.497	0.620
	Non-dornase	141	37.01 ± 51.01		
Collapse duration (days)	Dornase	132	4.94 ± 3.88	1.863	0.064
	Non-dornase	142	4.15 ± 3.14		
CVP	Dornase	132	2.93 ± 5.63	-2.348	0.020
	Non-dornase	78	4.87 ± 6.04		
SpO_2_/FiO_2_	Dornase	132	212.87 ± 101.81	-2.232	0.026
	Non-dornase	136	246.34 ± 140.08		
FiO₂ (%)	Dornase	108	51.88 ± 22.63	0.808	0.420
	Non-dornase	136	49.54 ± 22.41		
PEEP trends	Dornase	109	6.39 ± 2.81	1.412	0.160
	Non-dornase	105	5.85 ± 2.85		
PEEP changes	Dornase	96	1.90 ± 0.70	-0.465	0.642
	Non-dornase	93	1.95 ± 0.79		
ICU length of stay (days)	Dornase	131	25.63 ± 27.58	3.680	0.001
	Non-dornase	135	15.09 ± 18.37		
MRAS score before treatment	Dornase	132	8.86 ± 3.26	4.744	0.001
	Non-dornase	143	7.07 ± 3.01		
MRAS score after treatment	Dornase	132	2.79 ± 2.33	3.248	0.001
	Non-dornase	143	1.93 ± 2.05		
DNase days	Dornase	131	5.58 ± 7.29	----	----
	Non-dornase	0	-		
Change in MRAS	Dornase	132	6.08 ± 2.69	3.019	0.003
	Non-dornase	143	5.14 ± 2.45		

Demographic and clinical parameters in pediatric patients

Table 3 presents the distribution of demographic and clinical parameters in pediatric patients receiving dornase compared to those who did not. Gender distribution was similar between the groups (p = 0.652). However, airway status showed a significant difference (p = 0.001), with all patients in the dornase group having an artificial airway, whereas the non-dornase group included patients with endotracheal tubes (ETT) and tracheostomies. Dynamic compliance was significantly worse in the dornase group, with a higher proportion of patients experiencing severe or moderate reductions (p = 0.001). Secretion characteristics also varied, with differences in both secretion amount (p = 0.015) and secretion color (p = 0.014), where more dornase patients had thick or yellow secretions. Additionally, 28-day outcomes differed significantly (p = 0.001), with a higher proportion of non-dornase patients being discharged (69 vs. 17), while the dornase group had more ICU transfers (83 vs. 49) and a higher mortality rate (25 vs. 10). There was no significant difference in atelectasis type (p = 0.208) or collapse duration category (p = 0.077).

**Table 3:** Distribution of demographic and clinical parameters in dornase and non-dornase groups. ETT: endotracheal tube; HFNC: high-flow nasal cannula.

Variable	Category	Dornase	Non-dornase	Chi-square	P-value
Gender	Male	74	74	0.203	0.652
	Female	61	68		
	Total	135	142		
Airway status	Artificial airway	86	0	172.516	0.001
	None	49	57		
	ETT	0	79		
	Tracheostomy	0	7		
	Total	135	143		
ETT level	Neutral	76	59	7.429	0.115
	High	6	9		
	Deep	5	13		
	Right mainstem	1	2		
	Trachea	1	0		
	Total	89	83		
Dynamic compliance	Severely reduced (0.00-1.00)	44	28	29.512	0.001
	Moderately reduced (1.01-2.50)	41	22		
	Mildly reduced (2.51-5.00)	24	24		
	Normal (5.01-10.00)	4	18		
	Increased (10.01 and above)	4	9		
	Total	135	143		
Type of respiratory support	HFNC	86	81	3.495	0.479
	Mechanical ventilation	20	31		
	O_2 _device	13	15		
	Room air	12	8		
	Total	131	135		
Secretion amount before treatment	Small, thin	10	17	14.091	0.015
	Small, thick	26	27		
	Large, thin	2	1		
	Large, thick	38	23		
	Moderate, thin	8	1		
	Moderate, thick	43	60		
	Total	127	129		
Secretion color before treatment	White	93	88	12.497	0.014
	Clear	4	18		
	Yellow	21	16		
	Blood	4	5		
	Other	5	1		
	Total	127	128		
28-day outcome	Discharged	17	69	47.628	0.001
	Transferred to ward	83	49		
	Expired	25	10		
	Still in ICU	6	7		
	Re-admitted to ICU	0	1		
	Total	131	136		
Type of atelectasis collapse	Compressive	27	21	1.586	0.208
	Resorptive	105	122		
	Total	132	143		
Collapse duration category	1 week	110	131	5.138	0.077
	2 weeks	16	8		
	3 weeks or more	6	3		
	Total	132	142		

Correlation analysis

Table 4 presents the correlation matrix analyzing the relationships between clinical variables in pediatric patients with persistent atelectasis. Notably, collapse duration showed a weak but significant positive correlation with PEEP trends (r = 0.162, p = 0.018) and ICU length of stay (r = 0.179, p = 0.003), suggesting that longer collapse duration is associated with increased PEEP adjustments and prolonged ICU stays. The SpO_2_/FiO_2_ ratio, an indicator of oxygenation efficiency, had a strong negative correlation with FiO_2_ (r = -0.423, p = 0.000), indicating that as FiO_2_ increased, oxygenation efficiency decreased. Additionally, PEEP trends were positively correlated with both PEEP changes (r = 0.292, p = 0.000) and ICU length of stay (r = 0.283, p = 0.000), implying that patients requiring more frequent PEEP adjustments had prolonged ICU admissions. However, CVP (central venous pressure) did not show significant correlations with other key clinical parameters.

**Table 4:** Correlation matrix. * Indicates a significant correlation at the 0.05 level; ** indicates a highly significant correlation at the 0.01 level. CVP: central venous pressure; PEEP: positive end-expiratory pressure; SpO_2_: peripheral capillary oxygen saturation; FiO_2_: fraction of inspired oxygen; ICU: intensive care unit; Sig.: significant.

Correlation matrix		Collapse duration	CVP	SpO_2_/FiO_2_	FiO_2_	PEEP trends	PEEP changes	ICU (days)
Collapse duration	Pearson correlation	1	-.022	-.092	.036	.162^*^	.113	.179^**^
	Sig. (2-tailed)		.752	.134	.572	.018	.121	.003
CVP	Pearson correlation	-.022	1	.067	.111	-.007	.079	-.093
	Sig. (2-tailed)	.752		.337	.132	.922	.326	.180
SpO_2_/FiO_2_	Pearson correlation	-.092	.067	1	-.423^**^	-.119	-.143^*^	-.085
	Sig. (2-tailed)	.134	.337		.000	.084	.050	.165
FiO_2_	Pearson correlation	.036	.111	-.423^**^	1	.119	.138	-.005
	Sig. (2-tailed)	.572	.132	.000		.100	.068	.937
PEEP trends	Pearson correlation	.162^*^	-.007	-.119	.119	1	.292^**^	.283^**^
	Sig. (2-tailed)	.018	.922	.084	.100		.000	.000
PEEP changes	Pearson correlation	.113	.079	-.143^*^	.138	.292^**^	1	.035
	Sig. (2-tailed)	.121	.326	.050	.068	.000		.632
ICU days	Pearson correlation	.179^**^	-.093	-.085	-.005	.283^**^	.035	1
	Sig. (2-tailed)	.003	.180	.165	.937	.000	.632	

Multiple regression analysis to control variables

Table 5 shows a strong regression model (R = 0.992, R² = 0.984), indicating that 98.4% of the variance in persistent atelectasis is explained by the predictors. The high F-statistic (72.396, p = 0.000) confirms the model’s significance. The low standard error (4.307) suggests accurate predictions, highlighting the importance of these factors in patient outcomes.

**Table 5:** ANOVA regression analysis. Sig.: significant; Std.: standard; NA: not applicable; df: degree of freedom.

Summary and statistics	Model	R	R square	Adjusted R square	Std. error of the estimate
	1	0.992	0.984	0.970	4.307
	Sum of squares	df	Mean square	F	Sig.
Regression	37,595.017	28	1,342.679	72.396	0.000
Residual	612.031	33	18.546	NA	NA
Total	38,207.048	61	NA	NA	NA

The regression analysis in Table 6 examines the factors influencing ICU length of stay in pediatric patients with persistent atelectasis. The model identifies several key predictors that significantly impact patient outcomes. Among the clinical variables, airway status (B = 7.908, p = 0.007) is a significant positive predictor, suggesting that patients with compromised airways tend to have prolonged ICU stays. Additionally, central venous pressure (CVP) trends (B = -0.259, p = 0.045) show a significant negative association, indicating that fluctuations in CVP may influence ICU duration. Secretion color before treatment (B = -1.210, p = 0.040) is also a significant predictor, suggesting that certain secretion characteristics might be linked to shorter or longer ICU stays.

**Table 6:** Regression coefficients for ICU length of stay. ICU: intensive care unit; MRAS: Modified Radiological Assisted Score; DNase: deoxyribonuclease; ETT: endotracheal tube; CVP: central venous pressure; SpO_2_: peripheral oxygen saturation; FiO_2_: fraction of inspired oxygen; ​PEEP: positive end-expiratory pressure; Sig.: significance; Std.: standard. ICU length of stay (days) is a dependent variable.

Model (coefficients)	Unstandardized coefficients		Standardized coefficients	t	Sig.	95.0% Confidence Interval for B	
	B	Std. Error	Beta			Lower bound	Upper bound
(Constant)	-4.362	12.155	-	-.359	.722	-29.093	20.368
Age	.037	.021	.062	1.730	.093	-.006	.080
Gender	.419	1.667	.008	.251	.803	-2.972	3.809
Status	-1.471	1.536	-.028	-.957	.345	-4.596	1.655
Airway status	7.908	2.760	.078	2.865	.007	2.293	13.524
ETT level	-.197	1.046	-.005	-.188	.852	-2.326	1.932
Number of affected lobes	.281	.164	.049	1.709	.097	-.054	.615
Collapse duration (days)	-.210	.542	-.034	-.388	.700	-1.313	.892
CVP	-.259	.124	-.064	-2.086	.045	-.511	-.006
SpO_2_/FiO_2_	-.012	.018	-.042	-.662	.512	-.048	.025
FiO_2_ (%)	-.062	.063	-.057	-.973	.338	-.191	.067
PEEP trends	-1.338	.695	-.081	-1.925	.063	-2.752	.076
PEEP changes	1.302	1.003	.037	1.297	.204	-.740	3.343
Medications	-.051	.296	-.005	-.173	.864	-.653	.551
Secretion amount before treatment	-.481	.477	-.033	-1.007	.321	-1.452	.491
Secretion color before treatment	-1.210	.567	-.059	-2.136	.040	-2.363	-.057
Secretion amount after treatment	.087	.349	.007	.250	.804	-.622	.797
Secretion color after treatment	.993	.608	.049	1.633	.112	-.245	2.231
28-day outcome	1.020	1.231	.029	.829	.413	-1.484	3.525
Endotracheal tube size and cuff	-.153	.187	-.025	-.816	.420	-.534	.228
Background	-.172	.578	-.009	-.299	.767	-1.347	1.003
Type of atelectasis collapse	.719	2.177	.012	.330	.743	-3.709	5.147
MRAS score after treatment	-.785	.936	-.086	-.838	.408	-2.690	1.120
DNase days	.139	.132	.048	1.054	.300	-.130	.409
Pre MRAS	1.863	2.587	.059	.720	.477	-3.401	7.127
Post MRAS	1.963	2.845	.049	.690	.495	-3.825	7.752
Change MRAS	-.009	.616	-.001	-.014	.989	-1.262	1.245
Collapse duration category	2.633	3.458	.063	.762	.452	-4.402	9.668
ICU length stay category	7.225	.281	.955	25.704	.000	6.653	7.797

Notably, the ICU length of stay category (B = 7.225, p < 0.001) is the most significant factor, confirming its strong influence on prolonged hospitalization. However, other variables, including age, gender, FiO_2_ levels, PEEP changes, and post-treatment MRAS category, do not show statistical significance, implying that their impact on ICU duration is minimal. Overall, this analysis aligns with the primary objective of assessing prevalence and incidence while supporting the secondary objectives by identifying specific clinical and demographic factors associated with prolonged ICU stays.

## Discussion

The research examined both the prevalence and incidence of persistent atelectasis among PICU pediatric patients together with the therapeutic potential of dornase for its management. The point prevalence of persistent atelectasis reached 48.18% among patients who received dornase, while the non-dornase group experienced 51.82% atelectasis occurrence. The incidence analysis demonstrated a major distinction between groups: dornase-treated patients experienced 26.4 new at-risk patient cases, whereas non-dornase-treated patients developed 71.5 new cases. The significant gap between groups indicates the patients in the dornase group have a low incidence of persistent atelectasis in pediatric patients compared to the non-dornase group.

The research found significant variations in medical outcomes between these two patient groups. Patients in the dornase group experienced both reduced CVP and improved MRAS scores, which provided evidence to support dornase as an effective atelectasis management strategy. The patients who received dornase treatment spent longer periods in the ICU and experienced higher mortality rates, possibly because their conditions were more severe. The research also showed dornase treatment associated with worse respiratory conditions yet failed to demonstrate any direct relationship to improved survival rates. The dornase group experienced respiratory compromise partly because their airway status and secretion characteristics showed significant differences from the control group through thicker and yellow secretions. The findings are somehow similar to the already published literature. Terlizzi et al. (2022) carried out a review and found that deoxyribonuclease (DNase) was tested for 12 weeks in 320 cystic fibrosis (CF) patients with severe lung disease [11]. Both groups were similar in age, height, and weight. After treatment with DNase, the treated group had a larger rise in forced expiratory volume-1 (FEV1) (9.4% vs. 2.1%, p < 0.001) and forced vital capacity (FVC) (12.4% vs. 7.3%, p < 0.01) than the control group. There were no changes in antibiotic treatment days, hospitalization days, or adverse events across groups [11].

This prevalence study stated that the duration of atelectasis occurred together with increased ventilatory requirements, which led to longer periods of ICU treatment. A longer atelectasis duration resulted in a positive correlation with modified PEEP requirements, which in turn led to longer hospital stays for patients. The research demonstrates why adequate management techniques remain essential for preventing respiratory decline among children in critical care units. A study by den Hollander et al. (2022) found that dornase alfa led to a reduction in the duration of mechanical ventilation among children treated in an intensive care unit due to atelectasis [12].

The research outcomes of this study are compared with previous studies. Dornase therapy showed encouraging findings for pediatric persistent atelectasis patients in critical care as compared to previously published literature. In a previously published systematic review conducted by Claudius et al. (2015), it was found that there is no significant evidence of the effectiveness of dornase alfa in treating persistent atelectasis in pediatric patients in a critical care setting [13]. However, there are mixed results reported in already published literature, where some studies, such as Goetz et al. (2022), reported that dornase was efficacious in reducing secretions [14], while Claudius et al. (2015) reported limited or no significant efficacy in preventing secretions and symptom development [13]. The study's findings about dornase's impact on new atelectasis cases should be evaluated using a longitudinal research design that effectively reports atelectasis prevention strategies in pediatric patients, especially in critical care. The dornase group experienced longer ICU stays, which matches previous research showing intensive respiratory interventions lead to extended hospitalizations yet this duration might stem from patients in this group having more severe illnesses since their mortality rate was higher.

There are some strengths and limitations of this study. The study benefits from both its appropriate sample size and its robust statistical analysis that delivers comprehensive findings about atelectasis and ICU outcomes. The study analyzed multiple clinical and demographic variables to provide extensive knowledge about the multiple confounders that contribute to persistent atelectasis. The research study also contains some limitations. The non-randomized study design creates potential bias because the dornase and non-dornase groups might not match equally at their initial assessment. The statistical analysis accounted for some confounding variables yet residual bias might still exist. The research examined only short-term ICU outcomes but failed to assess respiratory health and quality of life after hospital discharge, which would have delivered a complete assessment of dornase's effectiveness.

Overall, the research demonstrates that dornase shows promise for treating persistent atelectasis in pediatric critical care by decreasing new case development. Additional research must validate these results while addressing the study's limitations, which include dornase's long-term effects and other clinical factors that affect atelectasis resolution.

## Conclusions

The research indicates that patients in the dornase group demonstrate a potential decrease in the incidence of new persistent atelectasis in pediatric critical care units. The non-dornase group showed slightly higher persistent atelectasis point prevalence and incidence rates. The study indicates that dornase can help lower the number of new patients with persistent atelectasis in critical care, but more research is needed to understand and determine causal relationships as well as the long-term effects of Dornase treatment and how it helps treat persistent atelectasis in seriously ill children.
